# Stakeholder Perspectives on the Structural Causes of Drug Shortages in Korea: A Mixed-Methods Study

**DOI:** 10.34172/ijhpm.9451

**Published:** 2026-03-15

**Authors:** Chungah Kim, Eunja Park, Dong-Sook Kim

**Affiliations:** ^1^Department of Preventive Medicine, College of Medicine, Chosun University, Gwangju, Republic of Korea.; ^2^Korea Institute for Health and Social Affairs, Sejong, Republic of Korea.; ^3^Department of Health Administration, Kongju National University, Gongju, Republic of Korea.

**Keywords:** Drug Shortage, Health Policy, Republic of Korea, Stakeholder Participation, Supply Chain Management

## Abstract

**Background:**

Drug shortages are a persistent global challenge. In South Korea, shortages occur within a distinct context characterized by high import dependence, inflexible pricing structures, and a hospital-centric distribution system. This study examined how frontline pharmacists and policy stakeholders perceive the structural causes of drug shortages across policy, supply-chain, and frontline pharmacy practice levels.

**Methods:**

We employed a sequential mixed-methods design to triangulate institutional perspectives with frontline realities, comprising six focus group interviews (FGIs) (n = 35) with policy stakeholders and a national web-based survey of 223 licensed pharmacists. Qualitative data explored institutional perspectives on structural causes and policy responses, whereas survey data quantified recent shortage experiences, impacts on patient care, and perceived causes.

**Results:**

Qualitative findings indicated that stakeholders attributed shortages to structural vulnerabilities, including unprofitable drug prices, dependence on imported raw materials, and distribution rigidities. These perceptions were supported by survey findings and aligned with frontline pharmacists’ reported experiences: 97.3% of pharmacists experienced shortages in the past three months, most frequently involving cold medicines and analgesics. Respondents identified raw material shortages (51.1%) and supply chain imbalances (17.5%) as primary drivers. Although pharmacists used coping strategies such as drug substitution, these adaptations were associated with substantial patient inconvenience (49.3%) and increased pharmacist workload (23%), indicating that individual-level responses are insufficient to address systemic failures.

**Conclusion:**

Drug shortages in Korea reflect systemic vulnerabilities compounded by distribution constraints rather than temporary disruptions. To address these perceived barriers, we propose a graded policy approach: short-term measures should prioritize administrative flexibility and information sharing, medium-term measures should focus on reforming pricing incentives, and long-term measures should strengthen supply chain sovereignty.

## Background

**Key Messages**

**Implications for policy makers**

- This study provides comprehensive insight into the perceived structural drivers of drug shortages, identifying economic disincentives, import dependence, and hospital-centric distribution rigidities as key factors.
- Current reliance on individual coping strategies (eg, ad hoc substitution) is unsustainable and may exacerbate system-wide instability. Policies should move beyond temporary fixes to address root causes.
- A graded approach to policy reform is recommended: prioritizing information sharing and administrative flexibility (short term), correcting unprofitable pricing structures (medium term), and investing in domestic manufacturing capacity (long term).

**Implications for the public**

Drug shortages can directly affect every day healthcare by limiting access to commonly used medicines such as cold remedies, painkillers, and injectable drugs. When these medicines are unavailable, patients may experience treatment delays, be asked to switch to unfamiliar alternatives, or face additional inconvenience and anxiety during already stressful health situations. This study shows that healthcare professionals in Korea perceive drug shortages as recurring problems linked to broader features of the pharmaceutical system, including pricing policies, reliance on imported raw materials, and distribution practices. Our findings help the public understand why shortages continue to occur and why individual pharmacies or hospitals often have limited ability to resolve them on their own. In the long term, policy efforts that support stable production of essential medicines, improve supply chain coordination, and strengthen monitoring systems may help ensure more reliable access to medicines and reduce disruption from future shortages.

Drug shortages are a persistent and serious global health challenge.^1,2^ For example, the United States reported over 300 active drug shortages in 2023, the highest level in nearly a decade.^3^ Similar trends have been observed globally, affecting essential medicines including oncology drugs, antibiotics, and sterile injectables.^4^ In South Korea, reported cases of supply disruptions increased by 60% between 2021 and 2024.^5^ Drug shortages prevent patients from receiving the right medications at the right time, with consequences that extend well beyond inconvenience. Such shortages can lead to delayed treatment, disease progression, complications, and treatment failure.^6^ They may also pose substantial patient safety risks, including an increased likelihood of medication errors and adverse drug reactions.^7^ Furthermore, patients may face considerable financial burdens due to higher treatment costs and unexpected out-of-pocket expenses.^8^ These challenges can also strain healthcare professionals by increasing workload and stress.^9,10^

 Drug shortages are a multifaceted problem arising from both supply- and demand-side factors, although research consistently points to supply-side disruptions as the primary driver.^11^ Shortages can emerge at any stage of the supply process—from raw material procurement to final distribution. Common causes include unreliable sources of active pharmaceutical ingredients(APIs),^12^ manufacturing issues such as facility shutdowns or quality control failures,^13^ and strategic business decisions by producers in response to low drug prices or insufficient demand.^14^ Additional contributors include voluntary recalls, product modifications, industry consolidation, and restricted distribution networks.^14^ Logistics challenges further complicate supply, particularly given strict requirements for temperature and humidity control during storage and transportation.^15^ Sudden demand spikes—such as those observed during pandemics—and external shocks such as natural disasters or geopolitical trade barriers can also destabilize the system.^15^

 Although global literature often emphasizes manufacturing issues and quality control failures, important gaps remain in understanding how these factors operate in Asian countries with distinct market structures. Qualitative studies from China suggest that national bidding and compulsory price-reduction policies may contribute to essential medicine shortages, although these policies are unlikely to be the sole cause.^16^ Similar economic pressures have been described in Japan, where repeated downward price revisions have been linked to recurrent shortages of low-profit generic products.^17,18^ However, empirical studies suggest that recent shortages in Japan were more directly associated with insufficient quality control and regulatory noncompliance than with price reductions alone.^17,18^ Collectively, these findings suggest that pricing policies may interact with other structural vulnerabilities rather than independently determining drug supply stability.

 Pricing pressures are frequently cited in qualitative studies and policy discussions; however, empirical evidence establishing a clear causal relationship between low medicine prices and drug shortages remains limited. A recent large-scale multinational study across 74 countries reported no significant association between medicine price levels and either the occurrence or intensity of drug shortages, indicating that pricing alone cannot explain supply disruptions.^19^

 Similarly, Korea’s reliance on imported raw materials and fragmented procurement structures creates distinct vulnerabilities that mirror regional economic disincentives. Existing research has primarily focused on supply-chain disruptions (eg, manufacturing delays, quality control failures)^20^ or demand shocks (eg, pandemics) as universal drivers of shortages.^14^ However, Korea’s experience highlights additional structural challenges related to how specific policies and market dynamics may translate into shortages: (1) Economic infeasibility: The Actual Transaction Price system mandates periodic price cuts based on market transaction data. This mechanism can erode profit margins for low-cost essential medicines and may discourage manufacturers from sustaining production, potentially contributing to market withdrawal.^21^ However, empirical evidence directly linking ATP reforms to observed shortages remains limited. (2) Supply chain fragility: Heavy reliance on imported APIs—primarily from China and India—limits domestic buffering capacity. Consequently, external shocks such as geopolitical conflicts or global logistics disruptions may, over time, translate into domestic supply interruptions.^22-24^ (3) Distribution and monitoring gaps: The pharmaceutical market is heavily influenced by large tertiary hospitals using competitive bidding, which can incentivize wholesalers to prioritize supply to these institutions to maintain contracts, creating “restricted networks” that marginalize community pharmacies. Furthermore, the lack of an integrated real-time monitoring system may prevent early detection of drug shortages and delay regulatory intervention until shortages become critical.^25^

 Despite these recognized challenges, empirical research remains limited in systematically capturing how structural vulnerabilities are experienced and understood by frontline healthcare professionals and institutional actors within Korea’s pharmaceutical system.^25^ Most existing studies rely on administrative data, media reports, or manufacturer-level information. However, institutional data alone may not capture the adaptations made by frontline professionals (eg, unauthorized substitutions or rationing) that buffer the system but may jeopardize safety. To address this gap, this study examined how stakeholders understand and navigate drug shortages across institutional and clinical contexts in Korea. Specifically, it aimed (1) to describe the current scope of drug shortages in Korea and their reported impacts on patient care, pharmacist workload, and healthcare organization management; (2) to identify and categorize stakeholder perceptions of the structural causes of drug shortages across external, supply-side, and demand-side domains, including pricing policies, import dependence, manufacturing and regulatory issues, and distribution constraints; and (3) to examine stakeholder-proposed policy and system-level responses to improve supply stability and resilience, such as adjustments to incentives for essential medicines, enhancements to information systems, and reforms in distribution and dispensing practices. Rather than asserting definitive causal relationships, the study aimed to generate empirically grounded, policy-relevant hypotheses for strengthening Korea’s pharmaceutical supply system.

## Methods

###  Study Design and Sampling

 This study employed a sequential mixed-methods design, beginning with qualitative focus group interviews (FGIs) followed by a quantitative web-based survey. The qualitative phase captured system-level perspectives on structural factors contributing to drug shortages and potential policy responses among key institutional stakeholders. Findings from the qualitative phase informed the development of the pharmacist survey instrument. In the second phase, we developed and administered a questionnaire to practicing pharmacists to quantify the recent frequency and types of drug shortages, perceived causes across the pharmaceutical supply chain, and impacts on patient care and pharmacy practice.

 Licensed pharmacists were selected for the quantitative phase as the primary survey population for several reasons. Pharmacists are responsible for procuring and dispensing medicines and therefore carry out a central linking role at the interface between wholesalers, manufacturers, prescribers, and patients. Unlike prescribers or distributors, pharmacists routinely manage stockouts across multiple therapeutic areas, negotiate substitutions with physicians, and communicate changes to patients.

###  Qualitative Study: Focus Group Interviews 

 Six FGIs were conducted between July and August 2023 across seven stakeholder groups; the government group participated in two sessions because of scheduling constraints.

 The number of FGIs was determined a priori to ensure coverage across key stakeholder groups involved in medicine pricing, manufacturing, distribution, and use, while remaining consistent with established qualitative research methodology. Empirical and methodological guidance suggests that core themes in focus group research are typically identifiable within a limited number of groups, with over 80% of themes emerging within two to three focus groups and approximately 90% within three to six groups; thematic saturation is commonly reached within four to eight groups, depending on study scope and heterogeneity.^26^ During analysis, thematic saturation was assessed iteratively. After the fifth and sixth FGIs, no substantively new themes emerged regarding core structural causes of drug shortages or proposed policy responses, suggesting that saturation had been achieved for the study’s analytic objectives.

 Participants were identified through professional associations, institutional affiliations, and expert networks to ensure representation of major stakeholder groups involved in medicine pricing, manufacturing, distribution, and use. To minimize researcher-driven selection bias, official invitation letters were sent to relevant professional associations and institutions, and each organization was asked to formally nominate one or more representatives with direct institutional experience and sector-level knowledge related to drug supply and policy. This nomination-based approach helped support balanced representation across stakeholder groups and reduced arbitrary participant selection by the research team.

 The final sample included representatives from pharmaceutical wholesalers; pharmaceutical manufacturers (domestic and multinational); medical and hospital associations; community and hospital pharmacies; patient and civic organizations; academia; and government agencies responsible for drug reimbursement, assessment, and supply management (Table 1). All participants provided written informed consent before participation.

**Table 1:** Characteristics of the Interviewees

**Group**	**Date**	**No. of Participants**	**Affiliation**
1 (Wholesalers)	Jul 12, 2023 (Wed)	Male 5	3 Persons from Korea Pharmaceutical Distribution Association, 2 Pharmaceutical Wholesalers
2 (Pharmaceutical Companies)	Jul 20, 2023 (Thu)	Male 4	Korea Pharmaceutical and Bio-Pharma Manufacturers Association
		Female 2	Multinational Pharmaceutical Company
3 (Hospital Association/Medical Association)	Jul 19, 2023 (Wed)	Male 3	2 Persons from the Korean Medical Association, 1 person from the Korean Hospital Association
4 (Pharmacies)	Jul 19, 2023 (Wed)	Female 3	Department of Pharmacy, Large Hospital
		Male 2	Korean Pharmaceutical Association
5 (Public–Patients/Civic Groups)	Jul 13, 2023 (Thu)	Male 1, Female 1	Patient Group
		Male 1, Female 2	Civic Group
6 (Academia)	Jul 13, 2023 (Thu)	Male 2, Female 2	College of Pharmacy Professor
7 (Government)	Aug 16, 2023 (Wed)	Male 1, Female 1	Rare Essential Medicines Center
	Aug 17, 2023 (Thu)	Male 1, Female 4	Health Insurance Review & Assessment Service Drug Information Center

 A semi-structured interview guide was developed using a primarily deductive framework informed by a review of international drug-shortage literature, Korean policy documents, and expert consultation; however, both the guide and the interview procedures allowed for inductive emergence of unanticipated themes. Interview topics included recent examples of drug shortages; clinical and operational consequences; perceived structural drivers (eg, raw material dependence, low pricing, forecasting failures, and regulatory audits); and stakeholder views on proposed policy interventions. Proposed interventions included government subsidies for essential drugs, establishment of a national pharmaceutical manufacturer, enhancements to real-time inventory and early warning systems, and reforms in distribution and prescribing practices.

 During analysis, we linked each proposed policy intervention to the structural causes of drug shortages discussed in the FGIs. For example, suggestions such as government subsidies or special reimbursement rules for essential low-margin drugs were described as responses to unprofitable drug prices, whereas proposals to strengthen real-time inventory and early warning systems were linked to information gaps and fragmented distribution. Similarly, ideas such as establishing a national pharmaceutical manufacturer or revising audit procedures were discussed in relation to vulnerabilities created by import dependence, limited domestic manufacturing capacity, and audit-triggered production halts. These articulated linkages informed our analytic framework and were used to construct and refine the quantitative survey instrument.

 As presented in Table S1 (Supplementary file 1), the interview procedure consisted of three stages: the preparation stage (initial contact and introduction of the study purpose and content), the interview stage (orientation, explanation of the study purpose and procedures, and discussion of research topics), and the closing stage (summary, debriefing, and immediate note-taking). All interviews were audio-recorded and fully transcribed.

 Transcripts were coded independently by two researchers using conventional content analysis within a hybrid directed framework. Before coding, we defined a set of sensitizing categories based on the interview guide (eg, pricing and reimbursement, manufacturing capacity, regulatory enforcement, distribution, and import dependence). Initial coding was conducted after repeated reading of the transcripts to achieve familiarity. Based on the initial codes, subcategories and broader categories were generated. When necessary, transcripts were revisited, and codes and categories were iteratively refined.

 During coding, coders were instructed to create new codes whenever meaningful segments did not fit the a priori categories. The codebook was updated iteratively as inductive codes and subthemes emerged (eg, “restricted networks” favoring tertiary hospitals, informal rationing practices, and “audit-triggered paralysis” of production), and earlier transcripts were revisited to ensure consistent application. Coding discrepancies were resolved through discussion until consensus was reached. The resulting thematic categories were used to construct and refine the quantitative survey.

 Building on the FGIs, we developed a web-based questionnaire for pharmacists by mapping qualitative themes onto survey categories (Table 2). For example, reports of frequent shortages of commonly used medicines informed items on the frequency and types of drug shortages and their impacts on dispensing practices and patient care, whereas discussions of unprofitable drug prices, dependence on imported raw materials, and distribution imbalances informed items assessing pharmacists’ perceptions of the underlying causes of shortages. Coping strategies described in the FGIs—such as substituting medicines, contacting prescribers to change prescriptions, relying on multiple wholesalers or manufacturers, and using online platforms to locate specific products—were translated into closed- and open-ended questions. To support content validity, the questionnaire was reviewed by health policy experts. Minor wording changes were made based on this feedback, but the overall structure and main question categories remained unchanged.

**Table 2:** Mapping of Qualitative Themes to Survey Questionnaire Categories

**Categories**	**Question**
Impact of drug shortages	How do drug shortages affect patient care? How do drug shortages affect hospital operations and management?
Causes of drug shortages	What do you think are the main factors contributing to drug supply shortages? Can you describe your experience with drug supply instability in your institution?
Solutions	Solutions for drug supply instability
	Solutions through central IT systems
	Opinions on government and pharmaceutical association policies
General characteristics	Characteristics of organizations
	Characteristics of individuals

Abbreviation: IT, information technology.

###  Quantitative Study: Web-Based Pharmacist Survey

####  Sampling and Participants

 A nonprobability snowball sampling method was used to recruit participants. Initial invitations were distributed to licensed pharmacists through national and regional pharmacist associations, and recipients were encouraged to forward the survey link to eligible colleagues within their professional networks. This approach was selected to efficiently reach pharmacists across diverse practice settings, including community pharmacies, hospital pharmacies, and hospital-based clinical pharmacy services. Because participation was voluntary and recruitment relied on network-based dissemination rather than a probability-based sampling frame, the geographic and demographic representativeness of respondents could not be systematically assessed. Furthermore, because recruitment occurred through secondary dissemination within professional networks, the recruitment denominator was not identifiable, and a conventional survey response rate could not be calculated.

####  Data Analysis

 A web-based survey was conducted in December 2023 with a total of 223 licensed pharmacists, including those working in community pharmacies, hospital pharmacies, and as hospital-based clinical pharmacists. Participants were recruited through snowball sampling, starting with contacts in national and regional pharmacist associations and professional networks. Snowball sampling was used because no comprehensive national list of practicing pharmacists with current contact information was available, and recent experiences of drug supply instability needed to be captured within a short time frame.

 Survey validity was addressed through a multistep process. The questionnaire was developed based on a review of prior literature on drug shortages and pharmacy practice to support content relevance. The draft survey was reviewed by subject-matter experts, including practicing pharmacists and researchers with expertise in pharmaceutical policy and supply chain issues, to assess face validity, clarity, and item appropriateness. A pilot test was conducted with a small group of licensed pharmacists to evaluate item comprehension, wording, and completion time, and minor revisions were made before full deployment. Although formal psychometric validation was not conducted, these procedures were implemented to strengthen the validity and reliability of the survey instrument.

 Quantitative responses were analyzed using descriptive statistics to summarize key variables, including the proportion of pharmacists reporting recent drug supply disruptions, the number and types of medicines with unstable supply, perceived main causes of shortages, and the prevalence of specific response strategies across community and hospital settings. Open-ended survey responses were analyzed using conventional content analysis to identify recurring themes. Two researchers independently coded the free-text responses and grouped related codes into thematic categories concerning drug supply instability, perceived causes, and coping strategies. Discrepancies were resolved through discussion until consensus was reached. The resulting themes were used to complement and contextualize the descriptive statistical findings.

####  1. Impact of Drug Supply Instability on Patient Care and Hospital Management

 Stakeholders consistently identified drug shortages as a major disruptor of routine clinical practice and a source of strain on the therapeutic relationship. In particular, hospital directors and pharmacists emphasized that abrupt substitutions with unfamiliar alternatives could undermine patient trust and impede shared decision-making.

 “*Doctors know they can prescribe other medications, but patients believe they won’t recover unless they take the original drug, which can undermine trust between the physician and patient” *(Hospital director).

 Shortages were also described as a growing burden on hospital management, particularly under diagnosis-related group (DRG) payment. When low-priced options are discontinued and only higher-priced substitutes remain, hospitals operating under fixed DRG reimbursement reported limited capacity to absorb increased acquisition costs.

 “*For individually listed drugs, reimbursement is set based on price… But in hospitals under the DRG payment system, the impact can be significant… only expensive options remain” *(Hospital director).

 From the perspective of hospital managers, drug shortages therefore translate not only into clinical inconvenience but also into financial pressure, particularly in DRG-based settings. In Korea, most medicines are reimbursed under a fee-for-service system through the National Health Insurance, whereas a fixed-payment system applies to certain patients covered by DRGs. Hospital administrators reported that shortages created operational challenges, citing cases in which shortages of low-cost medicines necessitated the use of higher-priced alternatives.

####  2. Key Causes of Drug Shortages

 Participants emphasized that drug shortages arise from multiple interacting factors rather than a single cause. Figure summarizes three broad domains: external issues, supply-side factors, and demand-side factors.

**Figure:**
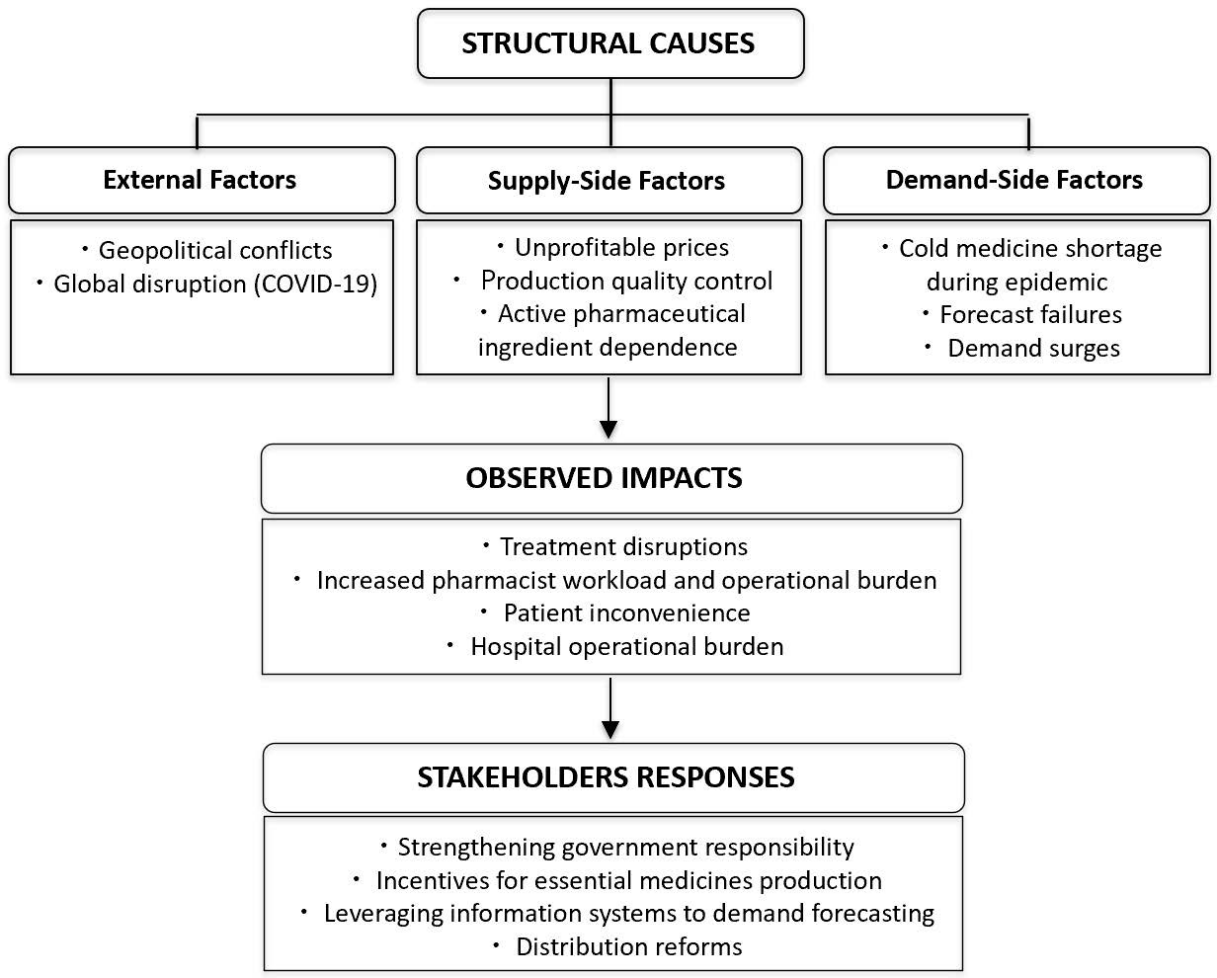
Themes Identified From Qualitative Analysis.

#####  2.1. External Issues

 Pharmaceutical manufacturing requires APIs, and shifts in global production have increased reliance on APIs manufactured overseas. Stakeholders identified geopolitical and other global events as key external drivers of raw material insecurity. The Russia–Ukraine war was cited as a concrete example of conflict-related disruption, and the COVID-19 pandemic was associated with widespread supply chain interruptions, lockdowns in supplier countries, and labor shortages in manufacturing lines. Participants also emphasized Korea’s heavy reliance on APIs imported from a small number of countries—particularly China and India—which they perceived as amplifying the impact of these shocks.

 “*Last year, the Russia-Ukraine war caused issues with raw material supply stability” *(Wholesale distributor).

 Academic and industry experts described the domestic supply as highly vulnerable because of Korea’s low self-sufficiency in pharmaceutical raw materials. They noted that accidents or shutdowns in foreign plants, fires at domestic facilities, and other unforeseen events could rapidly translate into national shortages because many manufacturers rely on a limited set of overseas suppliers.

 “*Korea’s self-sufficiency rate for raw materials is extremely low—we import nearly everything. If a foreign plant has an accident, the supply instability worsens” *(Academic expert).

 These accounts illustrate how globalization and concentration of manufacturing were perceived as limiting Korea’s capacity to mitigate supply disruptions.

#####  2.2. Supply-Side Factors

 Within the supply-side domain, three structural issues were repeatedly highlighted: unprofitable drug prices, production and quality control problems, and dependence on imported raw materials.

*a. Unprofitable drug prices: *Participants, particularly those from hospitals and industry, perceived unprofitable pricing as a major barrier to sustaining production and distribution of essential low-margin medicines. They suggested that thin profit margins weaken incentives to maintain continuous output and encourage batch production or market withdrawal.

 “*For low-priced essential drugs or those with thin profit margins, companies often produce them in bulk only once a year, and since there is a long gap before the next round of production, shortages can occur during that time” (Hospital director*).

 This quotation illustrates how frontline stakeholders linked pricing policies with extended production cycles and intermittent stockouts.

*b. Regulatory and quality control issues*: Technical failures, capacity limitations, and regulatory enforcement related to Good Manufacturing Practices were also identified as pivotal contributors to supply instability. Participants noted that when Good Manufacturing Practices violations are detected, regulators may suspend production or distribution for all products manufactured at a facility, jeopardizing supply across multiple product lines.

 “*If a drug is found to violate manufacturing safety standards, production or distribution is suspended. Korean pharmaceutical companies don’t just manufacture a single product—so even a one-month suspension affects all items they produce” *(Hospital pharmacist).

 Quality problems involving contaminated raw materials were also mentioned as triggers for withdrawals and recalls.

#####  2.3. Demand-Side Factors

 On the demand side, participants highlighted two interlinked themes: shortages of widely used medicines and failures in demand forecasting during sudden surges.

*a. Demand increase for cold medicines*: Across stakeholder groups, three prominent examples emerged as the most frequently and consistently cited cases of drug shortages, spanning pharmaceutical companies, distributors, pharmacists, civil society, and academic experts. Shortages or unstable supply of cold medicines and antipyretic analgesics—especially acetaminophen-based products (eg, Tylenol)—were repeatedly cited as the most representative example. The issue gained widespread attention during and after the COVID-19 pandemic, when public demand surged. A participant from a civil society group emphasized that:

 “*What ordinary consumers experienced was a supply shortage of essential household medications like Tylenol and other acetaminophen-based drugs, which were considered necessary to keep on hand for COVID-19 symptom relief and prevention” *(Civil society group).

*b. Failures in demand forecasting and sudden spikes: *Participants reported that companies sometimes failed to anticipate sharp increases in demand associated with epidemics or seasonal peaks, leading to delayed procurement of raw materials and subsequent shortages.

 “*Sometimes, companies fail to forecast demand accurately, so they don’t procure raw materials in time” *(Wholesale distributor).

 Hospital pharmacists linked these forecasting challenges to rising bed occupancy and higher overall medicine consumption, suggesting that both long-term planning gaps and acute demand spikes were perceived to contribute to shortages.

####  3. Challenges and Stakeholder-Proposed Responses

 Stakeholders proposed a range of policy responses that directly addressed the structural drivers identified above. These proposals clustered into three themes: strengthening governmental responsibility and incentives for essential medicines, leveraging information systems, and reforming distribution and dispensing practices.

#####  3.1. Strengthening Governmental Responsibility and Incentives for Essential Drugs

 Participants across academia, hospitals, pharmacy organizations, and civil society argued that relying solely on market forces is insufficient when production of low-profit but clinically important drugs becomes unsustainable. They recommended that the government more clearly define what constitutes a drug shortage, identify high-priority medicines (eg, high-utilization or high-impact products), and introduce targeted measures—such as price adjustments, cost coverage, or other incentives—to maintain supply. Some stakeholders proposed establishing a public or national pharmaceutical manufacturer to produce drugs that private companies avoid or to secure emergency stocks. At the same time, they emphasized that such an initiative would require careful scoping to avoid inefficient use of public resources.

 “*Government resources should be allocated where necessary, while market-based mechanisms should be left intact in cases where they function well. Excessive regulation with potentially adverse effects should be avoided. For issues not directly linked to patient safety or supply risks, existing practices may suffice. However, targeted prioritization is essential for high-risk areas” *(Academic expert).

#####  3.2. Leveraging Information Systems to Manage Demand and Improve Distribution

 A second set of proposals focused on using existing and improved information systems to manage demand and support more equitable distribution. The Drug Utilization Review system was frequently cited as a tool for implementing real-time prescription alerts, limiting duplicate prescriptions for shortage-affected drugs, and enforcing maximum prescription durations (eg, capping long-term prescriptions at 30 days and short-term prescriptions at 5–7 days). Stakeholders also called for strengthening the National Pharmaceutical Information Center so that inventory and distribution data could be monitored by region and supply chain level. They emphasized, however, that these systems would need to be designed with frontline usability in mind.

 “*The pharmaceutical information system should allow us to monitor inventory levels and distribution flows in real time, segmented by region and supply chain” *(Community pharmacist).

#####  3.3. Reforming Distribution and Dispensing Practices to Improve Responsiveness

 Finally, participants discussed reforms to distribution and dispensing practices. Hospital representatives expressed concern that mandatory substitution or active-ingredient-based prescribing could create conflicts between prescribers and pharmacists, whereas community pharmacists tended to support ingredient-based prescribing and emphasized the need to simplify legal procedures for interpharmacy transfers. Although current regulations allow pharmacy-to-pharmacy exchanges, the associated documentation and invoicing requirements were described as burdensome, leading some pharmacists to bypass formal procedures during acute shortages.

 “*Administrative procedures need to be simplified”*(Community pharmacist).

###  Quantitative Survey Results

 The number of medicines reported as having unstable supply varied across respondents; therefore, the percentages reported in Table 3 reflect the proportion of respondents to each item rather than the full sample of 223 pharmacists. Table 3 shows that among the 223 respondents, the largest age groups were those in their 30s and 50s, followed by those in their 40s and 60s. The most common specialties for which respondents dispensed medications were internal medicine, pediatrics, otolaryngology, and orthopedics. Most respondents worked in community pharmacies, with a smaller number affiliated with hospital pharmacies or working as hospital-based pharmacists.

**Table 3:** Survey Responses (% of Respondents to This Item)

**Categories**	**Variable**	**Respondents ** **(N = 223)**	
		**No.**	**%**
Age	20~29	7	3.1
	30~39	65	29.1
	40~49	52	23.3
	50~59	68	30.5
	60+	31	13.9
Experience of drug shortages in the past 3 months	Yes	217	97.3
	No	3	1.3
	No response	3	1.3
Impact of shortage on pharmacies and patients	Inconvenience to patients	110	49.3
	Increased pharmacist workload	51	23.0
	Patient distrust	51	23.0
	No effect	10	4.5
Number of medicines with unstable supply in the past 3 months	1 item	1	1.0
	2–4 items	25	11.2
	5–9 items	71	31.8
	No response	4	4.0
List of drugs by frequency of shortages, therapeutics (main ingredient)	Analgesics (acetaminophen)	108	16.9
	Nasal decongestants (pseudoephedrine)	101	15.7
	Osteoarthritis treatments (avocado–soy unsaponifiables)	86	13.4
	Laxative (lactulose)	60	9.3
	Inhaled corticosteroid (budesonide)	24	3.7
	Decongestant (pseudoephedrine + levocetirizine)	22	3.4
	Mucolytic agent & bronchodilators (ambroxol + clenbuterol)	20	3.1
	Type 2 diabetes mellitus (dulaglutide)	16	2.5
	Opioid analgesic (dihydrocodeine)	14	2.2
	Treatment of influenza (oseltamivir)	13	2.0
	Topical ointment (mupirocin)	10	1.6
	Nonsteroidal anti-inflammatory drug syrup (dexibuprofen)	10	1.6
Sources of drug shortage information	Pharmaceutical wholesalers	117	52.5
	Pharmacist networks	88	39.5
	Pharmaceutical companies	6	2.7
	Online wholesale malls	2	0.9
Main cause of shortage	Import issues of raw materials and other components	114	51.1
	Profitability	40	17.9
	Supply chain imbalance	39	17.5
	Regulatory action for deficiencies in manufacturing facility quality	16	7.2
	Legal issues such as rebate penalties	7	3.1
Action taken in response to drug shortages	Dispensed equivalent ingredients	84	37.7
	Could not dispense	63	28.3
	Called medical institution to change prescription	50	22.4
	Dispensed after it became available	25	11.2
Impact of drug shortages on pharmacists	Does not cause problems	1	0.4
	Causes problems	217	97.3
	Do not know	5	2.2

 Among the 223 respondents, most pharmacists (97.3%) reported experiencing drug supply disruptions in the past three months. The most commonly reported impacts were inconvenience to patients (49.3%), increased pharmacist workload (23.0%), and patient distrust (23.0%). Only 4.5% reported no impact. The main perceived causes of drug shortages were import-related raw material shortages (51.1%), supply chain imbalances (17.5%), and profitability issues (17.9%).

 Commonly reported medicines with unstable supply included acetaminophen-based analgesics (16.9%), nasal decongestants containing pseudoephedrine (15.7%), and selected injectable and chronic disease medications. Percentages presented in the table and text refer to item-level response frequencies and should be interpreted accordingly.

 When asked about the perceived main causes of supply disruptions in 2023, respondents most frequently identified raw material shortages and procurement challenges (51.1%), followed by distribution inefficiencies (17.5%) and regulatory delays (7.2%).

 In response to shortages, pharmacists reported several coping strategies, including dispensing therapeutically equivalent ingredients (37.7%), contacting prescribers to request prescription changes (22.4%), or being unable to dispense the prescribed medicine (28.3%). Although these strategies supported continuity of care in some cases, respondents emphasized that they increased workload and operational burden at the pharmacy level.

## Discussion

 This study provides new evidence on the scope, causes, and perceived policy solutions for drug shortages in South Korea. By integrating qualitative material from stakeholder discussions with survey data from frontline pharmacists, we show that drug shortages are not isolated incidents but rather a recurring, system-level feature of the current pharmaceutical supply system. Qualitative findings indicated that stakeholders attributed shortages to systemic factors such as economic disincentives and dependence on imported raw materials. These perceptions aligned with survey results, in which 97.3% of pharmacists reported experiencing supply disruptions and identified raw material shortages and supply chain imbalances as the main perceived causes. These findings reflect stakeholder perceptions and reported experiences rather than empirically verified causal mechanisms. However, although individual-level strategies such as drug substitution or sourcing through multiple platforms may provide temporary relief, they are insufficient to address the root causes of ongoing shortages.

 Previous research indicates that reliance on such improvised strategies is inherently unsustainable. These behaviors have been described as “beggar-thy-neighbor” tactics, in which aggressive sourcing or hoarding by one pharmacy directly depletes stock available to others.^27^ Rather than resolving underlying causes, these competitive behaviors may intensify system-level instability and undermine equitable distribution. Furthermore, this fragmented approach may exacerbate structural inequities within the pharmaceutical market. Recent empirical evidence suggests that pharmacies with greater purchasing power are more likely to secure inventory during shortages, leaving smaller community pharmacies and their patients disproportionately vulnerable and effectively creating a tiered system of access based on pharmacy size rather than patient need.^28^ Consequently, although these individual efforts may offer temporary local relief, they can also obscure the severity of upstream supply failures and delay adoption of necessary structural reforms.^27^

 Our findings are consistent with international literature that identifies low pricing and limited profitability as major contributors to drug shortages. A recent scoping review from the United States characterized shortages as chronic public health crises driven by economic and market failures.^2^ Similarly, a cross-national study across Austria, Germany, and Kosovo identified economic pressures—such as inflation and pricing constraints—as key drivers of supply instability.^29^ These findings also align with patterns observed in Western China, where shortages were frequently linked to market withdrawal of low-cost essential medicines, compounded by challenges in domestic production.^16^ However, large-scale empirical evidence suggests that the relationship between medicine price levels and drug shortages is not straightforward. A multinational analysis across 74 countries found no significant relationship between prices and either the occurrence or intensity of shortages, indicating that pricing alone is insufficient to explain supply disruptions.^19^ In addition, cross-national comparisons of generic drug prices indicate that Korea’s price levels vary substantially by therapeutic class relative to other high-income countries, suggesting that economic pressures on manufacturers are heterogeneous rather than uniformly low across the generic market.^30^ A similar trend was reported in Pakistan, where inflexible price controls and inefficiencies in drug procurement and distribution hindered consistent access to medicines, particularly in the public sector. Taken together, these findings suggest that pricing pressures may interact with other structural vulnerabilities rather than acting as an independent or dominant cause of shortages.

 However, the Korean context presents distinct structural challenges relative to these global patterns. Evidence from hospital-based studies in high-income settings shows that medication shortages remain recurrent and operationally demanding even in well-resourced systems. A five-year tertiary-care hospital study from the United Arab Emirates reported repeated shortages, with marked peaks during the COVID-19 pandemic, and emphasized the substantial administrative and coordination burden required to manage each event.^31^ These findings suggest that drug shortages are structurally embedded challenges rather than problems limited to resource-constrained contexts. However, limited research has examined how structural pricing and distribution systems shape frontline experiences in Asian health systems such as Korea. Our study addresses this gap by integrating institutional and frontline perspectives within a high-income, hospital-centric market.

 Beyond shared economic pressures, stakeholders emphasized additional vulnerabilities in governance and distribution. First, pandemic-related disruptions exposed long-standing structural weaknesses: without clear, unified national guidance, demand surges and utilization variability may place additional strain on limited supplies, underscoring the need for coordinated, evidence-informed protocols. Second, drug shortages appear to be shaped by overlapping regulatory and procurement challenges that, in Korea, are compounded by a market structure heavily skewed toward large tertiary hospitals that use competitive bidding systems.^32,33^ Wholesalers often operate under contracts that compel them to supply these institutions first to retain vendor status, which can result in the inventory being segmented into relatively isolated pools. This rigidity may limit the ability to redistribute medicines to community pharmacies during shortages, in contrast to more retail-oriented or decentralized systems in which inventory can move more readily in response to real-time demand. Finally, whereas some European countries have implemented early warning systems and centralized tracking mechanisms, Korea lacks similarly integrated infrastructure, making real-time coordination and response more difficult.^28^

 This study has limitations that indicate important directions for future research. First, a key limitation is the lack of triangulation with objective supply-side data. The present study captures stakeholder perceptions and interpretations of structural causes, which should be understood as perceived drivers rather than objectively verified causal relationships. Second, the purposive qualitative sample and nonprobability survey sample may limit generalizability and introduce selection bias. In addition, the nonprobability web-based survey distributed through pharmacist associations limits the ability to assess the representativeness of the 223 respondents in terms of geographic coverage and demographic characteristics. Third, self-reported data are subject to recall bias and reflect perceptions rather than objective verification. To bridge the gap between perception and reality, future studies should triangulate these findings with objective supply-side data, such as wholesaler inventory logs. Fourth, the cross-sectional design precludes causal inference. Longitudinal or intervention studies are needed to test whether specific policy reforms reduce shortage frequency over time—for example, by evaluating changes in stockout rates following implementation of targeted subsidies or real-time monitoring systems. Finally, these findings are specific to Korea’s hospital-centric bidding system; comparative research across regulatory environments may help isolate the effects of particular institutional arrangements on drug supply stability.

 To address the structural causes identified above, we propose a graded approach to policy reform categorized by feasibility and potential impact. In the short term, prioritizing administrative flexibility and information sharing offers high feasibility and potential for immediate impact. Specifically, leveraging the Drug Utilization Review system to manage demand during shortages and simplifying administrative procedures for interpharmacy transfers could provide near-term relief for the frontline workload by using existing infrastructure to reduce current inventory silos. In the medium term, improving the sustainability of essential low-margin medicines is important for market stability. Rather than assuming that price increases alone will prevent shortages, targeted measures such as differentiated pricing policies or subsidies for essential medicines may better align economic incentives with public health needs.

 Over the longer term, strengthening supply chain resilience by investing in domestic manufacturing capacity and reducing dependence on imported raw materials may provide a buffer against global supply shocks. These proposals should be interpreted as hypothesis-driven responses informed by stakeholder perspectives rather than definitive evidence-based solutions.

## Disclosure of artificial intelligence (AI) use

 Not applicable.

## Ethical issues

 The ethics committee at Gongju University approved the study protocol (Approval number: S-267/2021).

## Conflicts of interest

 Authors declare that they have no conflicts of interest.

## Supplementary files


Supplementary file 1 contains Table S1.
