# Supplementary material for: Stakeholder Perspectives on the Structural Causes of Drug Shortages in Korea: A Mixed-Methods Study
Source: Int J Health Policy Manag. 2026 Mar 15;15:9451. doi: 10.34172/ijhpm.9451 (PMC13145200; doi:10.34172/ijhpm.9451)
Supplement: Supplementary file 1 — contains Table S1. [file ijhpm-15-9451-s001.pdf]

Article title: Stakeholder Perspectives on the Structural Causes of Drug Shortages in Korea: A Mixed-Methods Study

Journal name: International Journal of Health Policy and Management (IJHPM)

Authors' information: Chungah Kim[1], Eunja Park[2], Dong-Sook Kim[3]*

[1]Department of Preventive Medicine, College of Medicine, Chosun University, Gwangju, Republic of Korea.

[2]Korea Institute for Health and Social Affairs, Sejong, Republic of Korea.

[3]Department of Health Administration, Kongju National University, Gongju, Republic of Korea.

***Correspondence to:** Dong-Sook Kim; Email: sttone@hanmail.net

Citation: Kim C, Park E, Kim DS. Stakeholder perspectives on the structural causes of drug shortages in Korea: a mixedmethods study. Int J Health Policy Manag. 2026;15:9451. doi:10.34172/ijhpm.9451

Supplementary file 1

Table S1. Process of focus group interview

Process		Contents
Preparation	Design	Finalize data collection methods and tools<br>Identify and recruit target groups (participants)<br>Decide schedule, time, and venue<br>Prepare interview question
	Initial Contact	Conduct introductory interview (warm-up)<br>- Introduce participants<br>- Build rapport<br>Confirm participants' demographic information
	Orientation	Explain research objectives and analysis direction<br>Explain interview schedule and procedure; check recording devices

Interview	Conduct interviews on research topics<br>- Ask structured questions<br>- Pose open-ended follow-up questions; facilitate opinion exchange
Closure	Conclude interview<br>Immediate note-taking (observations)<br>Organize recorded content<br>Analyze, interpret, and prepare report
